# Characteristic functional connectome related to Post-COVID-19 syndrome

**DOI:** 10.1038/s41598-024-54554-3

**Published:** 2024-02-29

**Authors:** Julia Bungenberg, Christian Hohenfeld, Ana S. Costa, Josephine Heine, Katia Schwichtenberg, Tim Hartung, Christiana Franke, Ferdinand Binkofski, Jörg B. Schulz, Carsten Finke, Kathrin Reetz

**Affiliations:** 1 Department of Neurology, RWTH Aachen University, Pauwelsstraße 30, 52074 Aachen, Germany; 2 JARA Brain Institute Molecular Neuroscience and Neuroimaging (INM-11), Research Centre Jülich and RWTH Aachen University, 52056 Aachen, Germany; 3 Department of Neurology, Charité-Universitätsmedizin Berlin, 10117 Berlin, Germany; 4 Faculty of Philosophy, Berlin School of Mind and Brain, Humboldt-Universität zu Berlin, 10117 Berlin, Germany; 5 Division for Clinical Cognitive Sciences, Department of Neurology, RWTH Aachen University, 52074 Aachen, Germany; 6 Institute for Neuroscience and Medicine (INM-4), Research Center Jülich GmbH, 52425 Jülich, Germany

**Keywords:** Neuroscience, Neurology

## Abstract

Post-COVID-19 syndrome is a serious complication following SARS-CoV-2 infection, characterized primarily by fatigue and cognitive complaints. Although first metabolic and structural imaging alterations in Post-COVID-19 syndrome have been identified, their functional consequences remain unknown. Thus, we explored the impact of Post-COVID-19 syndrome on the functional connectome of the brain providing a deeper understanding of pathophysiological mechanisms. In a cross-sectional observational study, resting-state functional magnetic resonance imaging data of 66 patients with Post-COVID-19 syndrome after mild infection (mean age 42.3 years, 57 female) and 57 healthy controls (mean age 42.1 years, 38 female) with a mean time of seven months after acute COVID-19 were analysed using a graph theoretical approach. Network features were quantified using measures including mean distance, nodal degree, betweenness and Katz centrality, and compared between both groups. Graph measures were correlated with clinical measures quantifying fatigue, cognitive function, affective symptoms and sleep disturbances. Alterations were mainly found in the brainstem, olfactory cortex, cingulate cortex, thalamus and cerebellum on average seven months after SARS-CoV-2 infection. Additionally, strong correlations between fatigue severity, cognitive functioning and daytime sleepiness from clinical scales and graph measures were observed. Our study confirms functional relevance of brain imaging changes in Post-COVID-19 syndrome as mediating factors for persistent symptoms and improves our pathophysiological understanding.

## Introduction

Post-COVID-19 syndrome is a poorly understood clinical condition that affects at least 10% of individuals with a prior SARS-CoV-2 infection leading to Coronavirus Disease 2019 (COVID-19)^1^. As biomarkers are still not available, the diagnosis is based on a temporal definition, defining long-COVID as symptoms persisting or newly developing four weeks and in case of Post-COVID-19 syndrome 12 weeks after SARS-CoV-2 infection^2^.

Besides respiratory complaints, Post-COVID-19 syndrome frequently includes neurological sequelae such as fatigue, cognitive impairment, smell and taste disturbances and headache^3,4^. These long-term symptoms have proven to be limitedly objectifiable by means of standard clinical diagnostic measures including routine clinical neuroimaging^5^. However, first in-depth metabolic, microstructural, and functional brain imaging studies have begun to uncover SARS-CoV-2 associated central nervous system (CNS) alterations that otherwise appear macrostructurally unremarkable in conventional brain imaging^6–11^. These studies have in common that multiple brain regions across distinct brain networks are affected and even if the extent of affected brain regions partly diverges between studies, a pattern of common CNS abnormalities after COVID-19 disease appears emergent. This pattern includes the olfactory cortex and associated regions, the thalamus, basal ganglia, the limbic system, the brainstem, and the cerebellum and was associated with clinical measures of fatigue and cognitive dysfunction^6–11^.

Despite emerging evidence from neuroimaging studies of virus-associated long-term imprints on the brain structure, literature characterising how they manifest in the functional connectome and whether they relate to clinical signs and symptoms is scarce^9,11,12^. One study found substantial network disruptions showing reduced connectivity between the left and right parahippocampal regions and the orbitofrontal and cerebellar areas 11 months after acute COVID-19 infection^9^. In contrast, Espoisto et al. found increased connectivity in the olfactory network, suggesting a compensatory response^12^. Our multicenter study aimed to uncover so far less-explored functional connectome changes post SARS-CoV-2 infection and assess their clinical relevance for commonly reported neurological symptoms in a cohort representing the majority of affected individuals, i.e. with mild acute infection. As distinctive features of our study, we used graph-theoretical measures in resting-state functional magnetic resonance imaging (rsfMRI) and employed both frequentist and Bayesian linear model analyses, providing compelling evidence of functional network alterations underlying neurological sequelae after mild initial COVID-19. Given the various neurological symptoms of Post-COVID-19 syndrome, we expected functional connectome alterations throughout brain areas rather than within specific networks. In particular, network disruptions in olfactory brain regions, and corticolimbic structures known to underlie executive dysfunction and fatigue in other neuropsychiatric diseases could be relevant in Post-COVID-19 syndrome as well.

## Results

### Study participants

Of the 123 included participants, 95 were female and 28 were male. The mean age of study participants was 42.2 years (standard deviation = 13.8). There was no difference in group membership (${\chi }^{2}\left(1\right)=0.316,p=0.574$) across sites, but there was evidence for a difference in distribution of sex (${\chi }^{2}\left(1\right)=5.675,p=0.017$) with a larger proportion of female participants in the patient group. There were no differences in age between sites (t(119.67) = 0.534,* p* = 0.594, 95% confidence interval [CI] for difference in means: [− 3.540, 6.158])). There was also no difference in average age between both groups ($t\left(94.864\right)=-0.065, p=0.949, 95\%\;{\text{CI}} \left[-5.298, 4.963\right]$).

### Clinical characteristics of the patient group

Time since infection was on average 7.03 months (SD = 3.7). The average total score of the HADS was 13.0 (SD = 7.6) with an average sub-score of 7.3 (SD = 4.2) for anxiety and 5.9 (SD = 4.3) for depression. The mean score for FSMC was 69.1 (SD = 18.4) indicating the presence of severe fatigue. The average total score for the PSQI was 8.1 (SD = 4.3) indicating impaired sleep quality. The ESS also indicated increased daytime sleepiness with an average score of 9.5 (SD = 5.8). The mean score for MoCA was 27.5 (SD = 1.9) indicating normal cognitive function. For full details on the sample and clinical characteristics details see Table 1.

**Table 1:** Sample and clinical characteristics.

Variable	Post-COVID-19	Healthy controls
Site Aachen/Berlin	38/28	29/28
Sex female/male	57/9	38/19
Age	42.3 ± 11.0; 41	42.1 ± 16.6; 41
Days since diagnosis	211.1 ± 107.0; 200	–
ESS	9.5 ± 5.8; 9.5	–
FSMC total	69.1 ± 18.4; 72.5	–
FSMC cognitive	33.9 ± 9.6; 36.5	–
FSMC motor	35.0 ± 9.6; 37	–
HADS total	13.0 ± 7.6; 11	–
HADS anxiety	7.3 ± 4.2; 6	–
HADS depression	5.9 ± 4.3; 5	–
MoCA	27.5 ± 1.9; 28	–
PSQI	8.1 ± 4.3; 7	–
TMT-A seconds	30.6 ± 11.6; 27	–
TMT-B seconds	66.7 ± 28.1; 57	–

Data is given as mean ± standard deviation and median, except for site and gender where counts are reported.

### Whole brain differences

On a whole-brain level, betweenness differed between groups ($p<.001,pd=1,\%ROPE=0$) with larger values in the Post-COVID-19 group. Weaker evidence was also observed for a difference in closeness with smaller values in the Post-COVID-19 group ($p=0.040,pd=0.987,\%ROPE=100$). For all other measures no differences were observed on a whole-brain level. Full details on whole-brain differences are given in Table 2.

**Table 2:** Global comparisons.

Variable	Estimate	pd	%ROPE	t-value	*p*-value
Mean distance	0.007	0.802	100	0.874	0.384
Diameter	0.130	0.939	36.079	1.578	0.117
Betweenness	0.491	1	0	4.190	< 0.001
Triangles	8.739	0.622	0.316	0.362	0.745
Katz Centrality	0.024	0.794	100	0.819	0.418
Hubness	–0.003	0.866	100	–1.099	0.272
Closeness	–0.003	0.987	100	–2.056	0.040

Shown are results for frequentist and Bayesian modelling. Abbreviations: pd: p-direction; %ROPE: percentage of values in region of practical equivalence.

### Regional differences

**Figure 1:**
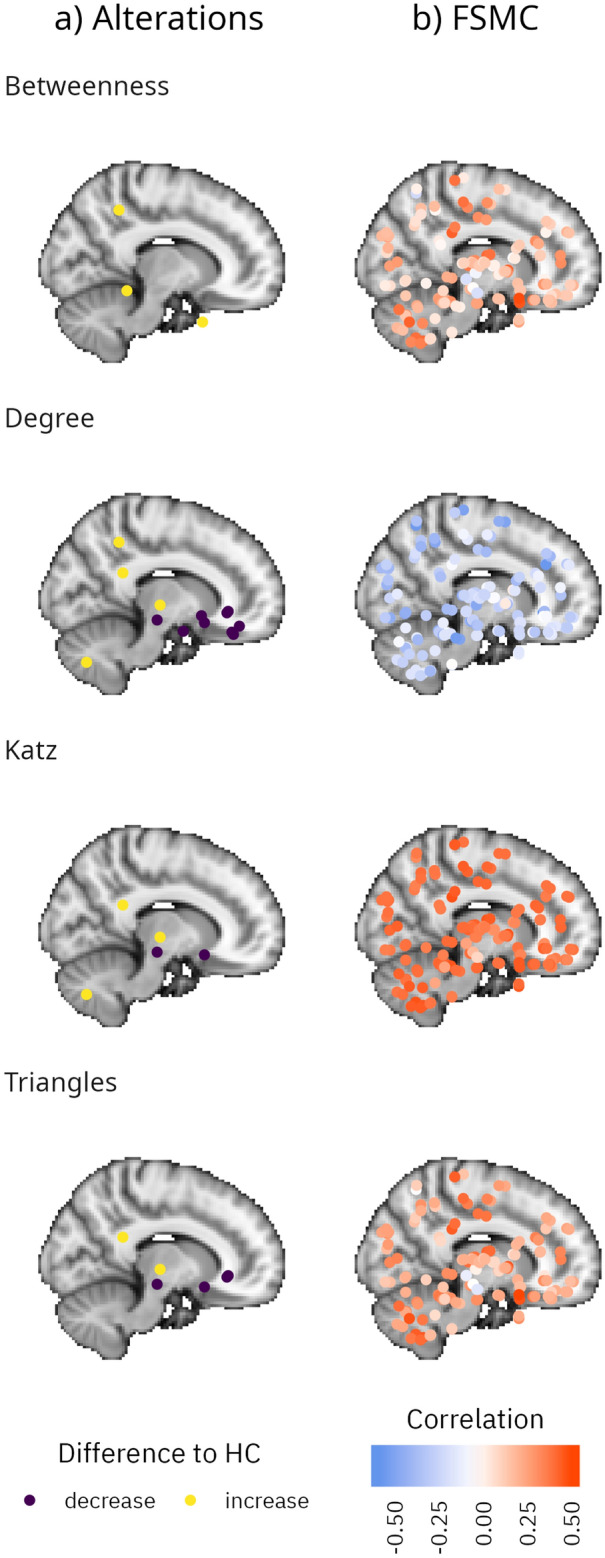
Spatial distribution of brain changes. Shown are differences between patients with Post-COVID-19 syndrome and controls (**a**) as well as Pearson’s correlations between graph measures and FSMC (**b**). Locations in the sagittal plane are mapped onto a single two-dimensional image. *HC* Healthy Controls.

Evidence for regional differences in the Post-COVID-19 group were found in the olfactory gyrus, cingulate cortex, red nucleus, thalamus and crus II of the cerebellum, among other regions. An overview of all regions and respective graph measures showing alterations is given in Table 3 and supplemental table S1, while a visualisation of the locations within the brain is depicted in Fig. 1.

**Table 3:** Regional comparisons including degree, betweenness, triangles and katz.

Region (degree)	Estimate	pd	%ROPE	t-value	*p*-value
Olfactory, right	− 14.552	0.998	0	− 3.022	0.003
Anterior cingulate sub, left	− 7.747	0.997	0	− 2.737	0.007
Posterior cingulate, left	6.978	0.995	1.2	2.577	0.011
Red nucleus, right	− 15.603	0.994	0	− 2.527	0.013
Anterior cingulate sub, right	− 8.82	0.993	0.692	− 2.493	0.014
Orbitofrontal cortex medial, left	− 11.53	0.989	0.226	− 2.359	0.02
Thalamus IL, right	6.194	0.983	5.824	2.113	0.037
Orbitofrontal Cortex lateral, left	− 9.841	0.983	2.318	− 2.159	0.033
Inferior parietal, left	4.783	0.982	9.432	2.149	0.034
Amygdala, right	− 6.789	0.981	5.171	− 2.084	0.039
Straight gyrus, right	− 7.452	0.98	4.553	− 2.083	0.039
Accumbens, left	− 7.425	0.978	5.032	− 2.027	0.045
Crus II, right	5.22	0.974	9.571	1.982	0.05
Region (betweenness)					
Vermis III	3.343	0.986	1.797	2.277	0.025
Inferior parietal, left	2.247	0.984	5.721	2.184	0.031
Medial temporal Pole, left	2.729	0.983	4.145	2.123	0.036
Region (triangles)					
Red nucleus, right	− 958.638	0.995	0	− 2.593	0.011
Olfactory, right	− 902.25	0.994	0	− 2.555	0.012
Posterior cingulate, left	619.243	0.989	2.545	2.358	0.02
Thalamus IL, right	631.901	0.989	2.332	2.375	0.019
Anterior cingulate sub, left	− 587.187	0.984	4.468	− 2.172	0.032
Anterior cingulate sub, right	− 637.012	0.983	4.403	− 2.111	0.037
Region (katz)					
Thalamus IL, right	0.55	0.992	2.158	2.414	0.017
Posterior cingulate, left	0.609	0.991	1.521	2.416	0.017
Red nucleus, right	− 0.816	0.99	0.816	− 2.305	0.023
Crus II, right	0.548	0.982	4.668	2.136	0.035
Olfactory, right	− 0.685	0.98	3.626	− 2.041	0.043

Shown are results for frequentist and Bayesian modelling. Estimate: Estimate from Bayesian analysis pd: p-direction; %ROPE: percentage in region of practical equivalence; sub: subgenual; IL: intralaminar.

### Correlations

Using the approach of setting a threshold based on the p-value associated with the t-test for a correlation different to 0, we found for fatigue (FSMC total score) a total of 64 regions (out of 142) with correlations exceeding that threshold in at least three graph measures. Regions were widespread with some focus on the cerebellum, temporal and occipital lobes, and thalamus. For affective symptoms (HADS total score) the threshold was only met in three regions, while for the daytime sleepiness (ESS) ten regions were involved, but for both measures no clear patterns emerged. Nine regions met the threshold for the PSQI, but without a clear pattern. For the TMT-A test, assessing cognitive processing speed, we found 19 regions meeting the criterion including the cingulate cortex, the insula and the paracentral lobule. Regarding the TMT-B, assessing task-switching capabilities, the criterion was only met in four regions.

**Figure 2:**
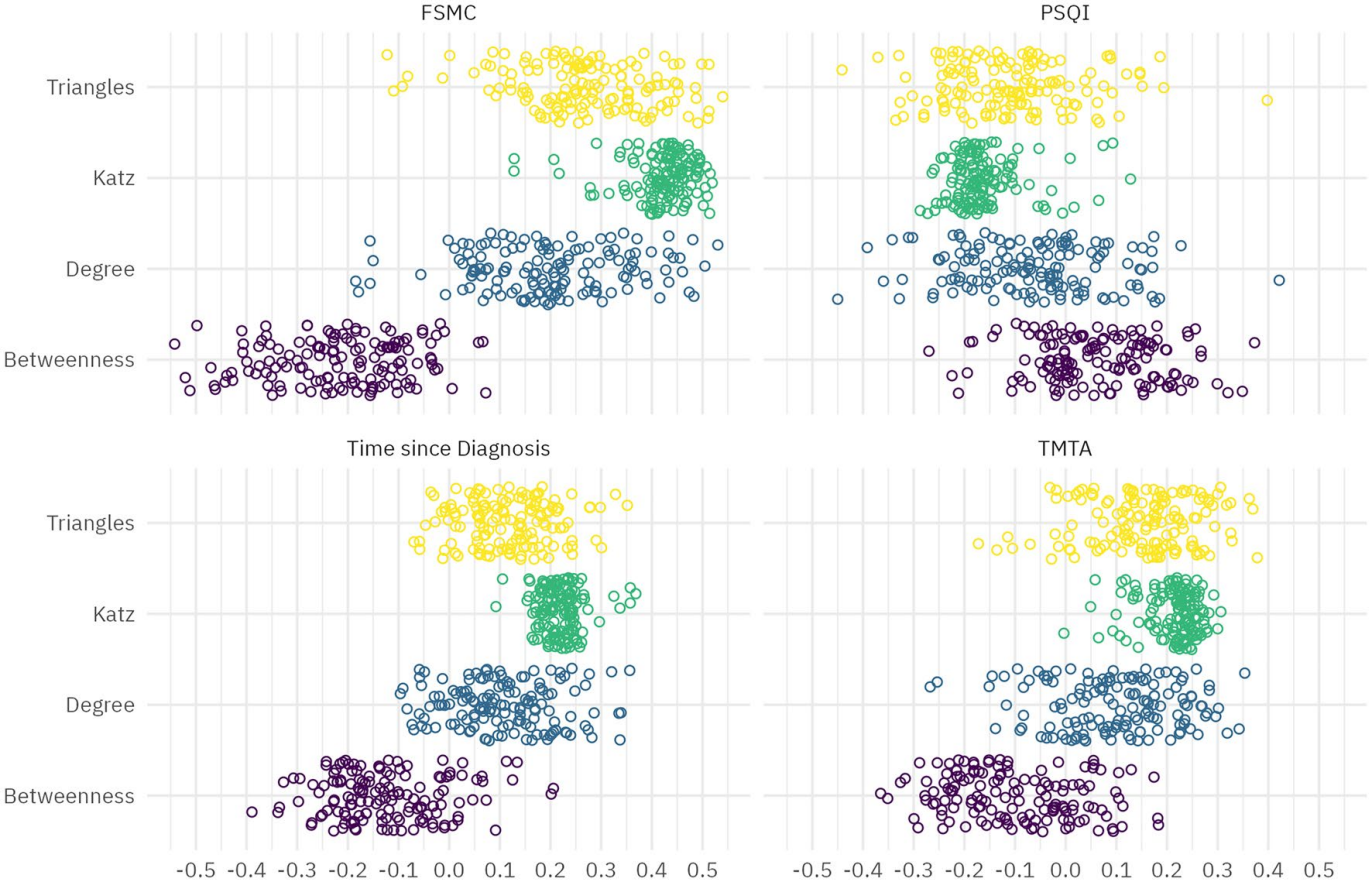
Distribution of correlations between selected clinical measures and graph data. Points indicate Pearson’s correlations coefficient between FSMC, PSQI, time since diagnosis and TMT-A and brain regions of respective graph measures including triangle, katz, degree and betweenness.

For the MoCA, the criterion was met in 12 regions, with a strong focus on the cerebellum and bilateral hippocampus. An equal number of correlations meeting the criterion was found for time since diagnosis, with some focus on the inferior frontal lobe and red nucleus, but with an overall more diffuse pattern. The distribution of correlations for most relevant measures is shown in Fig. 2 in which the points indicate Pearson’s correlations coefficient between FSMC, PSQI, time since diagnosis and TMT-A and brain regions of respective graph measures including triangle, katz, degree and betweenness. The spatial distribution of correlations between graph measures and FSMC score is visualised in panel b of Fig. 1. Regions where correlations of clinical variables and graph variables met the criterion of strong evidence and where there was evidence for a difference between groups are listed in Table 4.

**Table 4:** Overlap of correlations and group differences.

Region (days since diagnosis)	Graph measure	Pearson’s r	t-value	*p*-value	pd	%ROPE
Superior occipital, left	Closeness	0.281	2.325	0.023	0.98	0.019
Red nucleus, right	Closeness	0.297	2.467	0.016	0.986	0.004
Red nucleus, right	Degree	0.321	2.687	0.009	0.991	0
Red nucleus, right	Hubness	0.321	2.692	0.009	0.993	0
Red nucleus, right	Katz centrality	0.368	3.145	0.003	0.999	0
Red nucleus, right	Triangles	0.301	2.503	0.015	0.988	0.003
Region (FSMC)						
Inferior parietal, left	Betweenness	− 0.442	− 3.624	0.001	1	0
Crus II, right	Katz centrality	0.437	3.573	0.001	1	0
Posterior cingulate, left	Katz centrality	0.408	3.284	0.002	1	0
Olfactory, right	Katz centrality	0.377	2.994	0.004	0.998	0
Thalamus IL, right	Katz centrality	0.398	3.188	0.002	0.999	0
Region (MoCA)						
Crus II, right	Degree	0.432	2.667	0.012	0.99	0
Crus II, right	Hubness	0.369	2.209	0.035	0.979	0.022
Region (PSQI)						
Superior occipital, left	Closeness	− 0.337	− 2.345	0.024	0.982	0.011
Region (TMT-A)						
Posterior cingulate, left	Closeness	0.319	2.667	0.01	0.996	0
Inferior parietal, left	Closeness	0.243	1.984	0.052	0.971	0.057
Posterior cingulate, left	Degree	0.319	2.67	0.01	0.991	0
Angular, left	Hubness	0.304	2.529	0.014	0.989	0.003
Posterior cingulate, left	Hubness	0.331	2.78	0.007	0.998	0
Posterior cingulate, left	Katz centrality	0.3	2.496	0.015	0.994	0
Red nucleus, right	Katz centrality	0.255	2.09	0.041	0.975	0.041
Posterior cingulate, left	Triangles	0.369	3.154	0.002	0.997	0

All regions are shown where there was at least weak evidence for a difference between groups and pd for correlation unequal 0 was 0.97 or larger. pd: p-direction; %ROPE: percentage in region of practical equivalence; IL: intralaminar.

## Discussion

This study is among the first showing substantial changes in the functional connectome of the brain in on average seven months after acute COVID-19. We have observed widespread changes in network architecture including the brainstem, olfactory cortex, cingulate cortex, thalamus, orbitofrontal cortex, and the cerebellum in Post-COVID-19 patients when compared to age- and sex-matched healthy controls. Furthermore, changes in network architecture were related to clinically relevant Post-COVID-19 manifestations, including fatigue severity and cognitive dysfunction. Importantly, our study cohort represents the typical clinical phenotype of neurological Post-COVID-19 outpatient clinics, that is, primarily female, mid-aged patients with mild to moderate COVID-19 not requiring hospitalization^13^.

Up to date, there is accumulating evidence of brain changes following SARS-CoV-2 infection in the absence of macrostructural lesions, which include metabolic, structural and functional alterations. However, although a recurring pattern of brain regions including limbic structures, the primary and secondary olfactory cortex, thalamus, brainstem, and cerebellum have been repeatedly reported as altered after SARS-CoV-2 infection, there is still a great variety regarding their magnitude and clinical relevance^6–11^. There have been for example reports of increased, as well as decreased, regionally gray matter volumes in similar brain regions and timeframes in long-COVID patients partially correlating with measures of fatigue and cognitive dysfunction^6,9,10^. In addition, fatigue was found to be associated with microstructural and volumetric changes of the thalamus and basal ganglia^7^. Hyper- as well as hypometabolism, was found in acute and subacute stages of COVID-19, possibly in a time dependent manner^14,15^. Until know, there have been only few studies focusing on functional brain connectivity changes after COVID-19, which are warranted to clarify and confirm the functional significance of reported structural and metabolic brain abnormalities. Recently, Diéz-Circada and colleagues conducted a multimodal imaging study on 86 patients, but only 36 healthy control, 11 months after COVID-19^9^. They found substantial network disruptions, which manifested as reduced connectivity between the left and right parahippocampal regions and the orbitofrontal and cerebellar areas and that were accompanied by reduced grey matter volume in cortical, limbic and cerebellar areas in Post-COVID-19 patients. Markedly, they also describe significant associations between cognitive dysfunction and the loss of grey matter volume. These changes in both brain structure and cognitive function were more notable among hospitalized patients. In another study of non-hospitalized individuals, decreases mainly within and between temporal and subcortical regions, such as the thalamus, parahippocampal gyri, amygdala, basal ganglia, and superior temporal gyri, were associated with more Post-COVID-19 symptoms four to five months after infection compared to symptomatic individuals with non-COVID infection^16^.

Our study substantially advances previous research as it investigates a critical, therapy-relevant timepoint in the chronicity process of Post-COVID-19, notably three to four months after the subacute phase and focuses on the vast and, therefore, most relevant patient group, namely female patients with mild initial infection^3^. One region that emerged as significantly hypoconnected across most regional graph measures was the red nucleus. The robust correlation with the time since diagnosis could suggest its susceptibility as one of the earliest or most profoundly impacted regions, and/or its delayed recovery. Located in the midbrain of the human brainstem the red nucleus is a key component of the motor pathway that connects the cerebral cortex to the spinal cord.

Accordingly, in a multicenter study, 66 of 143 patients showed brainstem hypometabolism compared to healthy study participants^8^. Harboring neurons of respiratory and cardiovascular circuits, the reticular activation system as well as serotonergic, noradrenergic, and dopaminergic neurons, brainstem dysfunction overlaps with major Post-COVID-19 symptoms including cognitive impairment with executive attention in particular, fatigue, depression, anxiety, headache, myalgia and pain perception^17^. Although we found no such clinical correlation, our data support the idea that chronic brainstem dysfunction is implicated in Post-COVID-19 symptomatology, which should be addressed when exploring new therapeutic avenues.

We also found a decreased functional connectivity in the olfactory cortex and the medial orbital gyrus which forms the olfactory sulcus and is adjacent to the olfactory tract. As sudden loss of smell is a frequent and early symptom of acute COVID-19 our findings may reflect prolonged network disruptions originating from the initial infection^3^. However, olfactory disturbance has a high and early spontaneous remission rate pointing to network disruptions of either neglectable functional relevance or manifesting in higher-order cognitive processes related to connecting brain regions, such as memory relevant limbic brain structures. Indeed, the longitudinal landmark study of Douad et al., proposed a limbic olfactory network as the main disease pattern following COVID-19, in which anosmia-related deprivation of sensory input could potentially lead to longitudinal abnormalities in regions connected to the primary olfactory cortex. Specifically, they found greater changes in markers of tissue damage in regions that are functionally connected to the primary olfactory cortex in individuals infected with SARS-CoV-2 compared to uninfected controls. They also observed a greater reduction in grey matter thickness and tissue contrast in the orbitofrontal cortex and parahippocampal gyrus, which both have secondary connections to the olfactory cortex.

Repeatedly, brain imaging studies have reported abnormalities across distinct severity grades of acute infection, implicating the cingulate cortex, which is part of the limbic lobe^6,18–20^. It contains the cingulate gyrus and is subdivided in an anterior and posterior part. We found connectivity changes affecting the cingulate cortex as well including a significant decrease in connectivity of the anterior cingulate cortex. Considering its functional connection to the piriform cortex our finding adds further evidence to limbic olfactory dominated network perturbations following SARS-CoV-2 infection. By contrast, we found an increased connectivity of the left posterior cingulate gyrus, which is a central node of the default mode network. Hyperconnectivity in highly linked brain areas may impair default mode network suppression, which is crucial for complex cognitive tasks. Indeed, we found a strong association of the TMT-A, a test of attention, to hyperconnectivity of the posterior cingulate gyrus which could contribute to the deficits in executive functioning we described in Post-COVID-19 patients before^5^.

In the thalamus, we found increased connectivity of the intralaminar nuclei which also strongly correlated with reported fatigue symptoms. The intralaminar nuclei receive input from various regions of the brain, including the cortex, the basal ganglia, and the brainstem, and modulate wakefulness, attention, and the sleep–wake cycle via outputs to the cortex. Remarkably, Heine et al. recently identified structural correlates of self-reported fatigue, 7.5 months after COVID-19 infection, which included aberrant fractional anisotropy, shape deformations and decreased volumes of the thalamus and basal ganglia. Interestingly, these alterations overlapped with subcortical changes known from multiple sclerosis, in which fatigue is a dominant non-motor symptom^7^. Furthermore, diffusion markers correlated not only with fatigue severity, such as physical fatigue, and fatigue-related impairment in everyday life, but also with daytime sleepiness. By contrast investigating brain changes in long-COVID patients in a similar timeframe after acute infection Besteher et al. described significantly enlarged grey matter volumes in several clusters including basal ganglia and thalamus in both hemispheres when compared to controls, but no association with symptom burden also assessed by neuropsychiatric symptom questionnaires and MoCA^10^. For now, the reasons for these seemingly contradictory remain unclear, but they may, in any case, narrow the scope for targets of future imaging biomarkers.

Lastly, we also observed an increase of connectivity of the crus II of the right cerebellar hemisphere that strongly correlated with the MoCA, a measure of cognitive performance. The cerebellum's role in various cognitive functions and its 'cognitive topography' has been extensively described before^21–23^. As such language, working memory, and spatial processing have been localized to crus I and II^24^. Among the cerebellar lobules, lobule VIII and lobule VII, including crus II, outstand in their significant connections with association areas of the cerebral cortex linked to higher order behavior, including executive functioning known to be impaired in Post-COVID-19 syndrome. Intriguingly, grey matter reduction in particularly crus II, was also reported by Douad et al. and was associated with greater cognitive decline in COVID-19 infected individuals^25^. Similarly, cerebellar hypometabolism was also reported by others and found to be associated with olfactory symptoms, cognitive complaints, pain, and insomnia in a FDG-PET study of 35 long-COVID patients compared to healthy controls^8,26^.

Despite advancements in understanding how SARS-CoV-2 infection impacts the brain, the underlying pathomechanisms of long-term symptoms, ranging from chronic inflammation to direct viral-induced mechanisms, autoimmunity, viral re-activation and psychosomatic manifestations, remain hypothetical.

Elevated inflammatory markers eight months after COVID-19 infection indicate chronic inflammation as mediating factors potentially leading to brain impairment^27^. Interestingly, in a study with 54 rheumatoid arthritis patients, abnormal anterior cingulum connectivity was associated with higher peripheral inflammatory markers^28^. In the future, studies assessing the impact of systemic inflammation especially on neural networks are sparse but warranted to delineate directed and indirect effects of viral infection.

In the context of our findings, it is worth mentioning that the viral cell-entry receptor angiotensin-converting enzyme 2 is abundant in posterior cingulate cortex excitatory neurons, interneurons, and the cerebellum, potentially rendering these regions vulnerable to acute and long-term viral-induced brain damage^29^. Autopsy studies have demonstrated that COVID-19 may impact the brainstem, inducing inflammatory responses, viral invasion, and neurodegeneration although evidence of SARS-CoV-2 neurotrophism is low^30–32^.

Lastly, factors unrelated to viral infection including psychosocial pandemic effects, premorbid mental and psychosomatic disorders greatly impact Post-COVID-19 syndrome and could manifest in the functional connectome as well^33^. Given that psychiatric co-morbidities such as depression represent a substantial risk factor for Post-COVID-19 syndrome and are predominantly regarded as network-based disorders, they could also mediate chronic postinfectious symptoms.

One major strength of our study is the comparatively large sample size including an age- and sex-matched control group and the focus on mild COVID-19 cases, which represents the majority of affected individuals worldwide. Furthermore, we used state-of-the-art imaging procedures, elaborate statistical analyses in combination with systematic and standardized clinical screening measures allowing for relevant conclusions on radio-clinical associations. This work has some limitations as well. First, the ideal control group would consist of sex and age-matched individuals who had experienced a systemic viral infection at the same time to delineate long-term effects specifically related to COVID-19 from general post-infectious sequalae, which was however not available within the study. Second, inclusion of study participants from two different sites with partially distinct scanning protocols can affect the results. To this end, we ensured equal distribution of groups and sex across sites and also ensured that mean age was not different between both sites. We also conducted separate analysis for each site (data not shown), which showed indeed more effects in global and regional measures for the Berlin site. The mainly affected brain areas we discuss here, i.e. cerebellar, limbic and thalamic structures, were however significantly affected in both, separate and pooled analyses.

In brief, our study provides evidence for an altered functional connectome after SARS-CoV-2 infection related to fatigue severity and cognitive functioning. It adds further evidence to brain changes in brainstem, limbic, olfactory, thalamic and cerebellar structures, as contributing factors for frequently reported Post-COVID-19 symptoms and narrows the search for potential future diagnostic biomarkers and targeted neuropsychological interventions. Longitudinal clinical studies should closely monitor the further evolution of clinical and imaging findings as the physiological recovery from COVID-19 seems to extend well beyond the resolution of acute symptoms.

## Methods

### Study participants

Participants were examined at sites in Aachen and Berlin, in Germany. At the Aachen site patients were recruited at the Department of Neurology at University Hospital RWTH Aachen. From the Aachen site, healthy controls without a history of psychiatric and neurological diseases were available from other research projects originating from the pre-COVID-19 era using the same imaging protocol. At the Berlin site both, patients and healthy controls without a history of psychiatric and neurological diseases were prospectively recruited for this study at the Department of Neurology at the Charité Berlin. Healthy controls had no history of previous SARS-CoV-2 infection.

Patients were eligible for participation if they were at least 18 years of age, had persistent, primarily neurological symptoms after an infection with SARS-CoV-2 confirmed by reverse transcription polymerase chain reaction (PCR) of nasopharyngeal swab or the presence of antibodies against SARS-CoV-2 without previous vaccination. Exclusion criteria to undergo magnetic resonance imaging (MRI), included contraindications for the use of research MRI, such as metallic implants or claustrophobia. From the available data of 247 study participants, 123 study participants (66 patients, 57 controls) were included in the analysis (Supplementary Materials Fig. S1). All procedures were approved by the local ethics committees (“Ethikkommission an der Medizinischen Fakultät der RWTH Aachen” and “Ethikkommission der Charité—Universitätsmedizin Berlin”) and followed the Declaration of Helsinki (EK 192/20, EA2/007/21 [Berlin]). All individuals gave written informed consent before participating in the study.

### Procedures

#### Clinical measures

As previously described, patients were neurologically examined, and a thorough medical history was recorded^5,7^. For this study we focused on the following standardised measures administered at both sites: the Fatigue Scale for Motor and Cognitive Functions (FSMC), the Hospital Anxiety and Depression Scale (HADS), the Epworth Sleepiness Scale (ESS), the Pittsburgh Sleep Quality Index (PSQI). For the FSMC patients assess their agreement on 20 items on a 5-point Likert Scale^34^. The FSMC distinguishes between physical and cognitive symptoms (10 items each). The overall score and subscales include cut-offs for mild (≥ 43), moderate (≥ 53), and severe fatigue (≥ 63). For the HADS-D^35^, the following severity thresholds were considered for each subscale: ≤ 7 for normal, 8–10 for questionable, and ≥ 10 for increased. ESS and the PSQI were administered for measuring symptoms of sleep disorders. Regarding cognitive functioning, the Montreal Cognitive Assessment (MoCA) was used as a brief screening tool for mild cognitive function^36^. Additionally, raw scores of Trail-Making-Test (TMT) A and B were used as a measure of cognitive processing speed (Part A) and cognitive flexibility (Part B).

#### Imaging

At both sites imaging was performed using Siemens (Erlangen/Germany) Prisma scanners with 3T field strength. A high-resolution T1-weighted anatomical scan and a functional imaging sequence were carried out in the resting-state. For the functional scan, the lights were turned off and study participants were instructed to not think about anything in particular, but could keep their eyes opened. Scanning parameters are given in Table 5.

**Table 5:** Scanning parameters.

Parameter	Aachen		Berlin	
	T1	EPI	T1	Multi-band EPI
Scanner	Siemens prisma		Siemens prisma fit	
Field strength T	3		3	
Dimensions	208 × 288 × 288	64 × 64 × 36	191 × 215 × 200	104 × 104 × 72
Volumes	n/a	205	n/a	720
Voxel resolution mm	0.8	3.1 × 3.1 × 3.6	1	2
Echo time ms	2.36	30	2.64	37
Repetition time s	2.4	2.21	2.5	0.8
Flip angle degrees	9	90	8	52

If just one value is given for the voxel resolution this indicates isotropic voxels.

### Data analysis

#### Pre-processing of imaging data

We carried out pre-processing of imaging data using the FSL software package (https://fsl.fmrib.ox.ac.uk/) accessed through a wrapper package implemented in the R programming language (R Core Team 2022). Pre-processing included putting data to standard orientation and skull-stripping of anatomical data. Functional data was motion corrected and checked whether total movement relative to the temporal midpoint of the scan exceeded more than 1mm of translation or 1° of rotation. If motion exceeded those limits the study participant was removed from further analyses. Also, we checked whether there were movement spikes exceeding 0.5mm from volume-to-volume (total movement was calculated assuming the brain is a sphere with a radius of 50mm) and flagged affected volumes if present for later removal. However, if movement spikes were present in the lower value of 20 volumes or 5% of volumes, the study participant was excluded in further analysis (Supplementary Materials Fig. S1).

Next, we ran automatic segmentation of anatomical data into white matter, grey matter and cerebrospinal fluid. We then proceeded with B0 unwarping of the EPI data, followed by co-registration of functional data into standard space. For co-registration we first generated warp matrices for the functional data into standard space (using linear and non-linear registrations with FSL’s tools flirt and fnirt), which were subsequently combined with the warp matrices from B0 unwarping to create a functional image in the standard space. Subsequently we also translated the tissue probability maps for white matter and cerebrospinal fluid into standard space using the previously obtained matrices. The tissue probability maps were now binarised at a threshold of 0.95 (with values in [0, 1]) and these maps then used to extract time courses of white matter and cerebrospinal fluid from the functional data. We further extracted the global signal time course of the entire brain area in the functional data. Additionally, we performed slice scan-time correction. For the previously extracted motion parameters we generated the first and second temporal derivative, merged this data with the white matter, cerebrospinal fluid and global time courses as well as a constant term and regressed these time courses out of the data.

The resulting data was then parcellated according to a modified version of the AAL version 3.1 atlas. The atlas was modified so that regions that are smaller than 10 functional voxels were either merged with neighbouring regions, or in case of the Raphé nuclei and locus coeruleus which have no immediate neighbouring regions in the atlas, entirely removed. The number of regions in the modified atlas is 142, compared to 166 in the original one.

Next, time courses were extracted for all regions, bandpass filtered (limits: [0.01, 0.15] Hz) and then scaled and centred. Finally, the first three and all previously flagged time points were removed from the regional time courses.

#### Graph construction

Graph construction and all subsequent analysis steps were carried out using the R programming language. For graph operations we used the tidygraph package. The previous steps lead to the generation of a $t\times 142$ matrix per study participant, with columns representing regions and $t$ the number of volumes. For each study participant, a pairwise correlation matrix of the regional time courses was calculated using Pearson’s correlation coefficient. The lower triangle of the matrix without the diagonal was used for graph construction, with each region becoming a node and the correlation coefficients becoming weighted, but undirected, edges.

As many commonly used graph theoretical measures in the analysis of functional MRI data can only be interpreted in simple graphs that are not complete, a threshold for binarisation of edges was sought. In a first step, we searched the proportion of weakest edges that could be removed from each individual graph without the graph breaking-up into several components. Obtained values were transformed into percent ranks and all values with a percent rank of less than five were discarded and the corresponding 7 study participants removed from further analysis^37^. Of the remaining values, the lowest proportional threshold was applied as the threshold for binarisation for all study participants.

#### Analysis of network features

We calculated a set of measures characterising different features of the network to gain insight about differences in network topology between patients with Post-COVID-19 syndrome and healthy controls. These measures can be distinguished by whether they characterise a feature of the entire graph (*global* measure) or whether they characterise a feature of individual nodes in the graph (*local* measure).

Global measures included the diameter (the length of the longest geodesic [shortest path]) and the mean distance (the mean length of all geodesics in the network). Local measures included the nodal degree (number of connections a node has), Katz centrality (centrality taking into account not only neighbours, but also nodes that are further away), triangles (the count of triangular patterns a region is involved in), betweenness (the number of times geodesics pass through a node) and closeness (characterising the distance to all other nodes). For Katz centrality we chose the dampening-factor $\alpha$ as $0.9\left(1/{\lambda }_{max}\right)$, where ${\lambda }_{max}$ is the largest eigenvalue of the adjacency matrix.

#### Statistical analysis

We compared central tendency in the aforementioned measures between healthy controls and patients with Post-COVID-19 syndrome to determine differences between groups. For local measures, differences were calculated both on the group level as well as on nodal level.

Both the commonly used *frequentist* approach of a linear model with one factor of group, as well as a Bayesian linear model implemented using the rstanarm R-package were used. For the Bayesian linear model, we used weakly informative Gaussian priors and fitted the model using four Markov chains with 2000 iterations each of which the first 1000 were used for *burn-in* and discarded.

The frequentist p-value, the Bayesian p-direction (pd) and the percentage of values in the region of practical equivalence (%ROPE) were applied to evaluate the presence of group differences.

We assume effects to be likely existing if $p<0.05$, $pd>0.97$ and $\%ROPE<2.5$ and report two different levels of evidence regarding effects to accommodate the array of computed measures. The term *strong evidence* refers to all three employed values indicating an effect, while the term *weaker evidence* refers to a situation where only two of the three values indicate an effect. The latter cases are usually small, but nonetheless likely existing, effects.

Additionally, we calculated correlations between clinical variables and graph measures by calculating Pearson’s product-moment correlation between both measures and using a t-test for an assessment of the effect. We also included the Bayesian correlation as implemented in the R-package BayesFactor and computed the same metrics as for the group comparison. The same strategy as outlined above, with two of three measures being past the thresholds as *weak evidence* and three agreeing measures as *strong evidence* were used to evaluate effects.

### Supplementary Information

Supplementary Information.

## Data Availability

Deidentified imaging data are available on request from the corresponding author.

## Abbreviations

CI: Confidence interval
CNS: Central nervous system
COVID-19: Coronavirus disease 2019
ESS: Epworth sleepiness scale
FDG: Fluorodeoxyglucose
FSMC: Fatigue scale for motor and cognitive functions
MoCA: Montreal Cognitive Assessment
pd: p-direction
PSQI: Pittsburgh sleep quality index
SARS-CoV-2: Severe acute respiratory syndrome coronavirus type 2
SD: Standard deviation
TMT: Trail making test
%ROPE: Percentage of values in the region of practical equivalence
