# Supplementary material for: Characteristic functional connectome related to Post-COVID-19 syndrome
Source: Sci Rep. 2024 Feb 29;14:4997. doi: 10.1038/s41598-024-54554-3 (PMC10904373; doi:10.1038/s41598-024-54554-3)
Supplement: Supplementary file 1 — Supplementary Information. [file 41598_2024_54554_MOESM1_ESM.docx]

# Supplementary Materials

**Figure S1:** Flow chart of study participant selection. The flow chart depicts the reasons and number of excluded study participants from both sites.

**Table S1: Regional comparisons including hubness and closeness.** Shown are results for frequentist and Bayesian modelling. Estimate: Estimate from Bayesian analysis pd: p-direction; %ROPE: percentage in region of practical equivalence; sub: subgenual; IL: intralaminar

| Region (hubness) | Estimate | pd | %ROPE | t-value | p-value |
| --- | --- | --- | --- | --- | --- |
| Olfactory, right | -0.108 | 0.997 | 0 | -2.879 | 0.005 |
| Posterior Cingulate, left | 0.057 | 0.996 | 0 | 2.769 | 0.007 |
| Red Nucleus, right | -0.119 | 0.993 | 0 | -2.491 | 0.014 |
| Crus II, right | 0.046 | 0.992 | 3.342 | 2.428 | 0.017 |
| Anterior Cingulate sub, left | -0.054 | 0.992 | 1.739 | -2.451 | 0.016 |
| Inferior Parietal, left | 0.038 | 0.99 | 6.282 | 2.365 | 0.02 |
| Orbitofrontal Cortex medial, left | -0.088 | 0.989 | 0.353 | -2.319 | 0.022 |
| Anterior Cingulate sub, right | -0.064 | 0.989 | 1.566 | -2.312 | 0.022 |
| Thalamus IL, right | 0.05 | 0.986 | 4.066 | 2.249 | 0.026 |
| Precuneus, left | 0.017 | 0.981 | 44.592 | 2.131 | 0.035 |
| Orbitofrontal Cortex lateral, left | -0.074 | 0.98 | 2.913 | -2.087 | 0.039 |
| Angular, left | 0.047 | 0.978 | 6.629 | 2.035 | 0.044 |
| Straight Gyrus, right | -0.057 | 0.977 | 5.124 | -2.02 | 0.046 |
| **Region (Closeness)** |  |  |  |  |  |
| Olfactory, right | -0.053 | 0.998 | 0 | -2.919 | 0.004 |
| Anterior Cingulate sub, left | -0.034 | 0.996 | 0.018 | -2.645 | 0.009 |
| Red Nucleus, right | -0.054 | 0.995 | 0 | -2.669 | 0.009 |
| Anterior Cingulate sub, right | -0.036 | 0.992 | 0.742 | -2.458 | 0.015 |
| Posterior Cingulate, left | 0.029 | 0.989 | 3.053 | 2.328 | 0.022 |
| Amygdala, right | -0.032 | 0.986 | 3.287 | -2.187 | 0.031 |
| Thalamus IL, right | 0.031 | 0.986 | 3.379 | 2.263 | 0.025 |
| Inferior Parietal, left | 0.025 | 0.983 | 6.224 | 2.2 | 0.03 |
| Orbitofrontal Cortex medial, left | -0.039 | 0.983 | 2.458 | -2.163 | 0.032 |
| Superior Occipital, left | -0.032 | 0.982 | 4.342 | -2.117 | 0.036 |
| Orbitofrontal Cortex lateral, left | -0.035 | 0.975 | 4.808 | -2.002 | 0.048 |
